# Posttraumatic stress symptoms and postpartum anxiety among palestinian women: the mediating roles of self-esteem and social support

**DOI:** 10.1186/s12905-023-02567-x

**Published:** 2023-08-09

**Authors:** Dana Bdier, Fayez Mahamid, Vicky Fallon, Moath Amir

**Affiliations:** 1 Psychology and Counseling Dept, An-Najah National University, Nablus, Palestine; 2 University of Milano-Bicocca, Milan, Italy; 3 School of Psychology, University of Liverpool, Liverpool, UK; 4 Department of Medical Laboratory, Palestinian Ministry of Health, Thabet-Thabet Hospital, Tulkarm, Palestine

**Keywords:** Stressful live events, Postpartum anxiety, Self-esteem, Social support, Palestine

## Abstract

**Background:**

Women are estimated to develop several mental disorders during pregnancy and/or for up to a year postpartum, with anxiety and depression being the most common co-morbidities. Postpartum anxiety is less well studied compared with postpartum depression in the Palestinian context in terms of risk factors, mental health outcomes and protective factors.

**Purpose:**

The aim of the current study was to investigate whether self-esteem and social support mediated the association between posttraumatic stress symptoms and postpartum anxiety among Palestinian women.

**Methods:**

*Berlin Social Support Scales, Postpartum Specific Anxiety Scale, Impact of the Event Scale, and Rosenberg self-esteem scale* were administered to 408 Palestinian women recruited from health centers in northern of the West Banks/ Palestine using a convenience sample.

**Results:**

The findings of our study revealed that postpartum anxiety positively correlated with posttraumatic stress symptoms (r = .56, p < .01), and negatively correlated with social support (r = − .30, p < .01), and self-esteem (r = − .27, p < .05). Moreover, posttraumatic stress symptoms negatively correlated with social support (r = − .24, p < .01), and self-esteem (r = − .25, p < .01). Results of structural equation modeling (SEM) showed a good fit of the hypothesized model.

**Conclusions:**

Given this, it is recommended to conduct similar studies with diverse samples in the Palestinian society. It would also be useful for health professionals who work with Palestinian pregnant women (i.e., mental health providers, nurses, midwives, physicians) to assess self-esteem and social support in an effort to identify women who may be at greater risk of developing postpartum anxiety. It may also be worthwhile to develop and implement interventions during pregnancy which serve to enhance a women’s sense of self-esteem during this particularly stressful period.

## Theoretical background

The transition to motherhood is expected to be a very stressful situation, in which mothers can be exposed to unfamiliar expectations and demands that extend beyond labor and delivery [35]. Having a baby is considered as a significant transitional life event, especially for women having their first child, as it involves changes in relationships between couples and within families, and is commonly a cause of additional financial stress, even among households with relatively high incomes [37].

As a result of this, women are vulnerable to mental health disorders, mainly postpartum depression (PPD) and postpartum anxiety (PPA), which can be defined as an excessive worrying that occurs after having a baby or becoming a parent (Fallon et al., 2016). PPA demonstrates a greater prevalence rate than depression and appears to be under-recognized [36]. It is estimated that the incidence of PPA ranges from 3 to 43%, and 8.5% of postpartum mothers are estimated to experience one or more anxiety disorders [13, 18].

During the postpartum period, several factors were identified as predicted risk factors for PPA including being a young mother, having a cesarean delivery, having fear of death during delivery, having fear of the birth, less self-confidence for the delivery, family conflicts and lack of family support [8, 9, 15].

Palestinian mothers are expected to be more vulnerable to postpartum anxiety as they live in a military occupied country with prolonged traumatic experiences and a shortage of mental health services [25, 26]. In a recent study, the prevalence of PPD in Palestine (28%) was higher than in high income countries, and high parity and unplanned pregnancy were identified as potential risk factors for PPD [30]. Also, the quality of life was 21.53 (out of a maximum score of 30) among a sample of Palestinian women in the postpartum period, in which the quality of life found to be affected by refugee status, the loss of a relative due to occupation, standard of living, and wanted-ness of pregnancy [20, 22]. found that exposure to traumatic experiences among Palestinian women was positively and significantly associated with complications during pregnancy and increased postpartum anxiety symptoms [21, 29].

### Posttraumatic stress symptoms, Postpartum anxiety and self-esteem

According to previous literature, posttraumatic stress symptoms which can be defined as a mental health condition that happens when individuals experiences traumatic and stressful events. Some of the common symptoms of posttraumatic stress symptoms include re-experiencing, avoidance, and hyperarousal [7]. Posttraumatic stress symptoms associated with high levels of maternal mental health symptoms during the postpartum period [3, 38, 30, 26, 27]. However, self-esteem has also been found to be a protective factor against psychological impairments during the postpartum period, and low self-esteem is considered as a risk factor for postpartum depression [14, 39].

[12]found that high levels of childcare stress or general perceived stress predicted a higher risk of postpartum anxiety, while maternal self-esteem and partner support predicted a lower risk of comorbidity. In a longitudinal study that aimed to investigate the coping styles of Chinese pregnant women, the results revealed that self-esteem related to positive coping [38]. Moreover, [27] investigated the role of self-esteem, social support and age on postpartum depression and anxiety among Nigerian women, and it was found that both self-esteem and social support have a protective effect on postpartum depression [11]. found that social support and self-efficacy buffered the effect of postpartum anxiety among women in postpartum period.

### Traumatic life events, postpartum anxiety and social support

Social support can be defined as a network of family, friends, neighbors, and community members that are available in times of need to give psychological, physical, and financial help [17]. This network provides four major types of support: opportunities to consult and share with others, substantive or material support, support for one’s self-esteem, and a sense of belonging. It can be received from multiple, different sources such as family, spouse, friends, and colleagues [1, 2]. Social support is considered as a coping strategy in which the individual feels that he/she is appreciated and loved [25].

Prior research has demonstrated a consistent pattern of social support being a protective factor against the likelihood of exhibiting symptoms of postpartum mental health issues. For example, a systematic review that explored the main risk factors for postpartum anxiety among mothers in Arab countries, found low social and husband support, and posttraumatic stress symptoms were identified as important risk factors [5]. Also, women with greater levels of perceived stress were more likely to have sustained postpartum anxiety, while women reporting greater social support from their partners were less likely to have sustained anxiety, in which woman with high stress levels because of negative life events (including poor partner support) may experience more anxiety, and woman who perceives emotional and structural social support will be more able to cope with posttraumatic stress symptoms and may remove some of the stressors experienced during postpartum period [9]. In a study by [37]Indicated that traumatic events, lack of social support, and social health factors predicated significantly postpartum anxiety at six months postpartum among Australian women.

### Current study

#### Setting

In our study, we targeted postpartum women in the West Bank of Palestine during a difficult period characterized by many conflicts and political violence between Palestinians and Israelis. In the last months, the political conflict between Palestinians and Israelis intensified, as the West Bank of Palestine witnessed many incursions by Israeli soldiers and settlers, these incursions were accompanied by shooting and arresting a large number of Palestinians. Undoubtedly, these violent events have contributed significantly in increasing the level of traumatic life events, and deterioration of mental health among several groups in the Palestinian society. Therefore, it is expected that these ongoing and posttraumatic stress symptoms may increase the level of anxiety in general and postpartum anxiety among Palestinian women in particular; previous studies [7, 10, and 26] have shown that postpartum anxiety can be increased among women living in affected populations. The current study was designed to examine the effect of stressful events on postpartum women living in Palestine. Specifically, whether these events led to an increased level of postpartum anxiety among postpartum women, and what are the protective factors that may mitigate the effect of traumatic events related to political trauma.

This study is important as it is the first to examine the relationship between posttraumatic stress symptoms and postpartum anxiety, and whether self-esteem and social support mediate the relationship between posttraumatic stress symptoms (predictor) and postpartum anxiety (as an outcome variable) among Palestinian women. Most of the previous studies reviewed concentrate on exploring the relationship between posttraumatic stress symptoms and postpartum depression, but not postpartum anxiety [3, 8, 12, 28, 31].

Based on prior research [3–5, 8, 12, 27, 38] study hypotheses were defined as: posttraumatic stress symptoms would be positively associated with postpartum anxiety (H1); Second, self-esteem and social support would mediate the association between posttraumatic stress symptoms and postpartum anxiety among Palestinian women (H2).

## Methodology

### Participants

Participants were Palestinian women recruited from five health centers in the West Banks of Palestine using a convenience sample. The advertisements detailed voluntary participation for a study testing the association between posttraumatic stress symptoms and postpartum anxiety, allowing health providers to provide needed psychological interventions. The sample size for this study was calculated based on 95% CI and 5% margin of error by using the Raosoft software sample size calculator. Based on that, the recommended sample was 408 women (see Table 1). The participants were Palestinian women of whom 65% were of ages 20–30 years and 35% percent ages 31–40 years. Regarding the age of newborns, 72% of women had children aged 1–60 days and 28% percent of women had children aged 61–120 days. 41.2% of participants had 1–2 children and 61.8% had 3–5 children. For Inclusion in our study, the participants are required to be: (1) Palestinian, (2) Native Arabic speakers, and (3) participants were mothers to infants between ages of 1 day to 6 months.

**Table 1:** Table 1: Demographic characteristics of study sample (N = 408)

Characteristic	Number	(%)
*Women’s Age*		
20–30 years	265	65
31–40 years	143	35
Total	408	100.0
*Children age*		
1–60 days	294	72
61–120 days	114	28
Total	408	100.0
*Number of children*		
1–2	168	41.2
3–5	240	61.8
Total	408	100.0

### Measures

All items of our scales were translated and back translated by English and Arabic language experts. The translated version of the scales was reviewed for Comprehensiveness and clarity by a panel of experts in psychological counseling, psychology, social work and health sciences. Finally, we pilot-tested the scales by distributing them to 80 participants (validity sample), and further modifications were made due to the validity and reliability indicators that we found.

*Berlin Social Support Scales (BSSS)*: The BSSS is a self-report scale developed by [32] to test several aspects of social support, including perceived social support, the need of support, support seeking, and provided support. The measure was primarily designed to test social support provided by families to their members who suffer from cancer; later on, it was used to test several types of social support among clinical and non-clinical groups. Participants respond to the scale using a four-point Likert scale, ranging from totally agree (4) to strongly disagree (1). The scale indicated a high level of reliability in assessing social support in the Palestinian context (α = 0.88).

*The Postpartum Specific Anxiety Scale (PSAS)*: The scale was developed by [13] and assesses anxiety symptoms pertinent to the postpartum period. The scale contains 51 items with four sub-scales: attachment anxieties and maternal competence, the items (1–15) represent this subscale. Welfare anxieties and infant safety, the items (16‐26) represent this sub-scale. Practical infant care anxieties, the items (27–33) represent this sub-scale. Motherhood and psychological adjustment, the items (34–51) represent this sub-scale. The responses to the items are interpreted on a 4‐point Likert scale ranging from 1 to 4 (1 = never, 2 = sometimes, 3 = often, 4 = almost always).

*The Impact of the Event Scale (IES-R)*: The IES-R is a self-report scale developed by [36]to test traumatic reactions in response to several traumatic events. The measure consisted of 22 items comprise three subscales: avoidance, intrusions, and hyperarousal. The intrusions sub-scale represents items related to nightmares, intrusive thoughts and memories, intrusive imagery and feelings related to traumatic events. The avoidance subscale represents items related to avoid places, people, and things related to traumatic experiences the person has had. The hyperarousal subscale represents items related to anger and irritability, difficult concentration, and psychophysiological arousal when exposure to reminders of traumatic experiences. The scale indicated a high level of reliability in the Palestinian context (α = 0.92).

*The Rosenberg self-esteem scale (RSES)*: The RSES is a self-report developed by [33] to measure self-esteem in general population. The scale consists of ten items, five items have positively worded, and five items were negatively worded. By assessing positive and negative feelings about self, the scale measures global self-esteem among respondents. Participants respond to the scale using a four-point Likert scale, ranging from very high (5) to very low (1). The scale indicated a high level of reliability in assessing social support in the Palestinian context (α = 0.88).

### Procedures

This study was conducted in May 2022 during political conflicts between Palestinians and Israelis in the West Bank of Palestine, and targeted Palestinian women with newborn infants. The sample was recruited from health centers in north of Palestine using convenience sampling techniques. Participants were provided with information to make an informed decision regarding participation in the study followed by a signed informed consent. Additionally, participants were informed on the purpose of the research and a brief description of the study instruments. Our study was approved by An-Najah National University Institutional Review Board (IRB).

### Data analysis

**Fig. 1:**
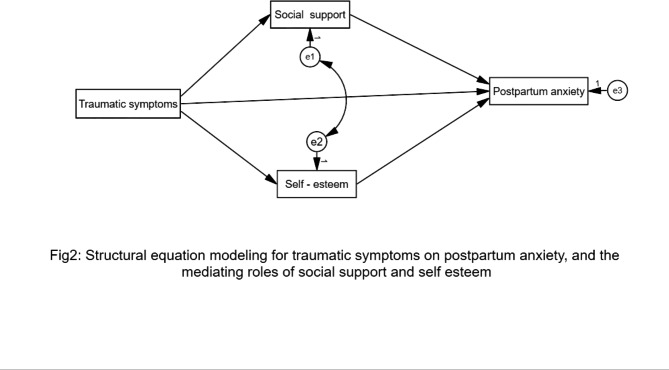
The conceptualized effect of posttraumatic symptoms on postpartum anxiety, and the mediating roles of social support and self esteem

Structural equation modeling (SEM) was conducted to test the conceptual model of our study, where posttraumatic stress symptoms were identified as a predictor variable, self-esteem and social support as mediators, and postpartum anxiety an outcome variable. We also calculated descriptive statistics for our study variables. Moreover Person Correlation Coefficient was used to test the significance of correlations among study variables. Finally, normed fit index (NFI), non-normed fit index (NNFI), root mean square error of approximation (RMSEA), standardized root mean square (SRMR), and comparative fit index (CFI) were tested. The thresholds for good fit were as follow: RMSEA < 0.041 and SRMR < 0.05, NFI > 0.94, CFI > 0.95. Lastly, we set a *P* value at 0.001. We tested our conceptual model (Fig. 1) using AMOS25 software for data analysis.

## Findings

Descriptive statistics related to the postpartum anxiety, posttraumatic stress symptoms, social support, and self-esteem were calculated as shown in Table 2. Participants reported high scores on social support and self-esteem. Moreover, participants scored moderate scores on postpartum anxiety and posttraumatic stress symptoms. Regarding reliability, all scales showed high reliability values ranging from 0.86 (*self-esteem*) to 0.95 (*postpartum anxiety*).

**Table 2:** Descriptive statistics for research variables (N = 408)

Variable	Mean	S.D	Min	Max	Range	Skewness	Kurtosis	Reliability
Postpartum anxiety	2.28	0.57	1.08	3.59	2.51	− 0.05	− 0.41	0.95
Posttraumatic stress symptoms	2.97	0.85	1.00	4.73	3.73	− 0.15	− 0.54	0.92
Social support	3.29	0.60	1.50	4.88	3.38	− 0.43	0.48	0.88
Self -esteem	3.27	0.74	2.60	4.20	3.30	1.48	1.08	0.86

Results of the correlational analysis are reported in Table 3. Namely, postpartum anxiety positively correlated with posttraumatic stress symptoms (r = .56, *p* < .01), and negatively correlated with social support (r = − .30, *p* < .01), and self-esteem (r = − .27, *p* < .05). Moreover, posttraumatic stress symptoms negatively correlated with social support (r = − .24, *p* < .01), and self-esteem (r = − .25, *p* < .01). Finally, a positive correlation was found between social support and self-esteem (r = .35, *p < .01*).

**Table 3:** Correlations among study variables (N = 408)

Measures	*1*	*2*	*3*	*4*
1.Postpartum anxiety	1	0.56**	− 0.30**	− 0.27**
2. Posttraumatic stress symptoms		1	− 0.24**	− 0.25**
3.Social support			1	0.35**
4.Self esteem				1

### Structural equation model (SEM)

**Fig. 2:**
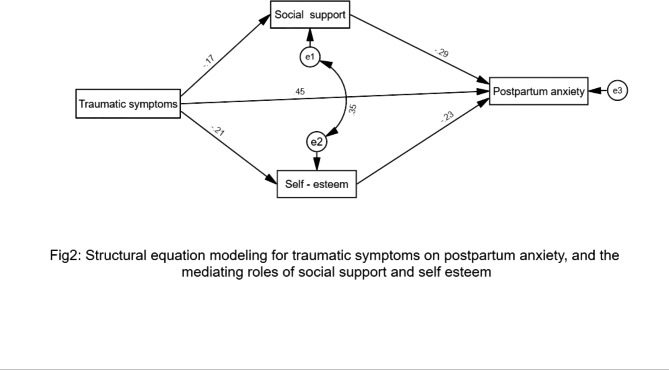
Structural equation modeling for posttraumatic symptoms on postpartum anxiety, and the mediating roles of social support and self esteem

Results of path analysis (Fig. 2) with posttraumatic stress symptoms as a predictor, social support and self-esteem as mediating variables, and postpartum anxiety as an outcome variable was tested across the sample (n = 408). Findings revealed that self-esteem and social support mediated the correlation between posttraumatic stress symptoms and postpartum anxiety with a good fit for the data (χ^2^_(4)_ = 138.25; *p* = .001; GFI = 0.93; AGFI = 0.94; RMSEA = 0.05; NFI = 0.93; CFI = 0.94).

Regarding (H1), analysis of path between posttraumatic stress symptoms, social support and self-esteem indicated a negative effect; social support (β_X,Y_ = - .-22 ; *p* < .001 and self-esteem (βX,Y = − 0.36 ; *p* < .001), a positive effect in path analysis was also found between posttraumatic stress symptoms and postpartum anxiety (βX,Y = 0.58 ; *p < .001*). Analysis of the path between social support, self-esteem and postpartum anxiety suggested negative effects; social support (β_M,Y_ = − 0.11; *p* < .001), anxiety(βM,Y = − 0.23; *p < .001*), (H2).

In regard to mediation hypothesis (H3), our model revealed a standardized total effect of social support (β_X,M_ = − 0.32; *p* < .001). However, this effect consisted of a statistically significant indirect effect (via social support, β_X,M, Y_ = -. 14 *p* < .01) and a statistically significant direct effect (β_X,Y,M_ = − 0.18; *p* < .01). The model also revealed a standardized total effect of self-esteem (βX,M = − 0.37; *p < .001*), this effect was consisted of a statistically significant indirect effect (via self-esteem, βX,M, Y = -. 16; *p < .01*) and a statistically significant direct effect (βX,Y,M = − 0.21; *p < .01*). Our results indicated that the association between posttraumatic stress symptoms and postpartum anxiety was mediated by social support and self-esteem. We also tested the effect of demographic variables in our model, and the results showed no significant differences due to these variables.

## Discussion

The current study was designed to test the association between posttraumatic stress symptoms and postpartum anxiety among Palestinian women, and whether social support and self-esteem mediated the association between the two variables. Our findings revealed a positive correlation between posttraumatic stress symptoms and postpartum anxiety, while a negative correlation was found between posttraumatic stress symptoms, social support and self-esteem. Moreover, a negative correlation was found between social support, self-esteem and postpartum anxiety. Finally, social support and self-esteem both mediated the correlation between posttraumatic stress symptoms and postpartum anxiety.

One possible explanation of our results is that Palestinian women with newborn infants experience the same as the majority of Palestinian people who are exposed to ongoing traumatic experiences due to the ongoing political conflict. This leads to a very negative impact on mental health, and may increase postpartum anxiety among those women [34]. In the Palestinian context, as elsewhere, childbearing and motherhood are closely interlinked with prevalent cultural beliefs and practices. Typically, female relatives engage in initiating new mothers into motherhood, from breastfeeding to bathing and physically caring for the child, giving advice, and managing other aspects of health and wellbeing and caring for the family. With increasing restrictions to mobility in Palestine due to geopolitical conflict and financial reasons, there is a possibility that this support system changed, especially as more young couples are leaving their homes and seek employment in different places, this could mean that new Palestinian mothers should cope with motherhood in isolation from the traditional female relative support system, which may lead to increase stressors and postpartum anxiety among new Palestinian mothers [20]. Also, the high levels of unemployment and unstable jobs among Palestinians, specifically for workers in the Israeli settlements could affect the mental health of new mothers as the low financial status of the family found to be associated with postpartum depression among Palestinian women [5]. Exposure to war trauma had impacts on postpartum anxiety among Palestinian women; [30]found that prevalence of postpartum depression in Palestine appears to be higher than in high income countries. Political conflict, unemployment and unplanned pregnancy were identified as risk factors for postpartum depression. Several international studies have supported our findings that high level of traumatic and stressful experiences experienced by pregnant and women with newborn infants are positively associated with postpartum anxiety, for example: [10]found that posttraumatic stress disorders among Turkish women were associated with postpartum anxiety, complications during birth, and fear of childbirth [21].found that women with PTSD symptoms had a worse quality of life and postpartum anxiety at postpartum weeks 4–6.

Social support and self– esteem significantly influence a woman’s ability to recover during the postpartum period [4, 6, 24]. Therefore, social support may serve as a protective factor against postpartum anxiety, specifically when women experience high levels of stressful and traumatic experiences, thus serving as a mediator. In regard to our findings, the high level of social support that women usually receive in the Palestinian society through family members, friends and partners in general, has contributed greatly in dealing with psychological and traumatic events experienced by the Palestinian women. Thus, it contributed to reducing the level of postpartum anxiety among those women [4]. found that social support was a significant protective factor against postpartum anxiety, with a high level of social support significantly mediated the correlation between posttraumatic stress symptoms and anxiety symptoms. Similarity, [6]found a significant correlation between low levels of family supports and postpartum anxiety.

Our findings revealed that Palestinian women with a high level of self-esteem could deal more effectively with stressful events, which may lead them to suffer less from anxiety during the postpartum period. This indicates that self-esteem could be considered as a protective factor that may mitigate the effect of traumatic and stressful events experienced by those women due to sociopolitical factors. An alternative explanation is that women with high level of self-esteem are more satisfied with their roles and responsibilities as mothers after childbirth, which will help them use positive coping strategies to deal with stressors and new challenges.

[16] found that self-esteem mediated the correlation between religiosity and postpartum anxiety among women in the United States [23]. found that self-esteem appeared to be a reliable contributing factor against depression and anxiety in the early postpartum period [19].found that self-esteem and social resources mediated the effects of stressors on mothers’ postpartum depressive symptoms, mothers with low self-esteem were 39 times more likely to have high depressive symptoms than those with high self-esteem.

### Limitations of the study

The current study has some limitations that may offer opportunities for future research to continue testing the correlation between postpartum anxiety and other related variables in the Palestinian context. First, the study used a convenience sample targeted Palestinian mothers who visited health centers in the north of Palestine. Evaluating the correlation between posttraumatic stress symptoms and postpartum anxiety and the mediating role of self-esteem and social support among different samples of Palestinian women who may at risk of developing postpartum anxiety and related mental health distress is crucial; the SEM model may differ in other populations. Secondly, we targeted postpartum women in the West Bank of Palestine during a difficult period characterized by many conflicts and political violence between Palestinians and Israelis. Hence, the political violence heightened feelings of general anxiety among individuals, possibly skewing the SEM findings. More future studies are recommended to test the correlation between current study variables over different periods of time. Thirdly, our sample is not sufficiently representative of at-risk women such as mothers experiencing domestic violence, women living in Palestinian internally displaced camps, and mothers of disabled children, indicating the need to evaluate the model of our study with at‐risk populations. Finally, our study used IES-R, RSES, and BSSS scales to test posttraumatic stress symptoms, self-esteem and social support; the psychometric properties of these measures were not tested within the Arabic language in a Palestinian context, indicating the need to validate these measures in a Palestinian context to increase the validity of the results.

## Conclusion

The current study aimed to test the correlation between stressful live events and postpartum anxiety, and whether self-esteem and social support mediating the correlation between the two variables. To our knowledge, this is the first study testing the current model in the Palestinian context. The findings of our study revealed a good fit of our model, where stressful live events operated as a predictor variable, self-esteem and social support as mediating variables, and finally postpartum anxiety was administered as an outcome variable. It is recommended to conduct similar studies with diverse samples in the Palestinian society; it would be prudent to target clinical populations which are needed to test the correlation between these variables and other variables related to women’s mental health in postpartum period. Given this, it may be useful for health professionals who work with pregnant women (i.e., mental health providers, nurses, midwives, physicians) to assess self-esteem and social support in an effort to identify women who may be at greater risk of developing postpartum anxiety. It may also be worthwhile to develop and implement interventions during pregnancy which serve to enhance a women’s sense of self-esteem during this particularly stressful period.

## Data Availability

The datasets generated during and/or analyzed during the current study are available from the corresponding author on reasonable request.
